# Spatially fractionated radiotherapy and immunotherapy: a synergistic strategy for systemic anti-tumor immunity

**DOI:** 10.1186/s12967-026-08548-6

**Published:** 2026-07-08

**Authors:** MengYao Li, Zhihui Dou, Shuting Wang, Xiaoying Fan, Maojie Tang, Tianyuan Dai, Yong Yin

**Affiliations:** 1 Department of Oncology, Affiliated Hospital of Southwest Medical University, Luzhou, China; 2 Department of Radiation Oncology Physics and Technology, Shandong Cancer Hospital and Institute, Shandong First Medical University and Shandong Academy of Medical Sciences, Jinan, China

**Keywords:** SFRT, Immunotherapy, TME, Abscopal effect, Bystander effect, Immune checkpoint inhibitors

## Abstract

**Purpose of review:**

This review summarizes preclinical evidence on the immunomodulatory mechanisms of spatially fractionated radiotherapy (SFRT) alone and in combination with immune checkpoint inhibitors (ICIs), as well as clinical evidence for SFRT with or without immunotherapy.

**Main body:**

Conventional radiotherapy (CRT) has a dual impact on anti-tumor immunity, simultaneously activating immune responses and inducing immunosuppression. Preclinical studies show that SFRT can remodel the tumor microenvironment (TME) and tumor vasculature, enhance effector immune cell infiltration, regulate cytokine networks, and elicit both bystander and abscopal effects. However, the magnitude and direction of these effects vary substantially across SFRT modalities, parameters, and tumor types.When combined with ICIs, SFRT shows promising tumor-control activity in preclinical models, yet it does not consistently outperform CRT plus ICI. Clinical evidence is still limited to small palliative cohorts and case reports without systematic immune monitoring. Nonetheless, isolated cases have reported abscopal responses and reversal of ICI resistance, highlighting the need for prospective, biomarker‑driven studies to define the immunologic and clinical role of SFRT.

**Conclusions:**

Preclinical evidence suggests that SFRT can induce both bystander and abscopal effects. These effects may help overcome immune resistance when SFRT is combined with immunotherapy, ultimately improving the therapeutic ratio of radiation and enhancing the efficacy of ICIs. However, current clinical data are still preliminary, and further systematic research is needed to optimize SFRT parameters, treatment sequencing, and tumor-specific responses.

## Introduction

Radiotherapy (RT) is the cornerstone of comprehensive cancer treatment [1]. Preclinical studies have demonstrated that the therapeutic efficacy of RT is derived not only from direct DNA damage but also from the activation of anti-tumor immune responses [2–4]. The immune-modulatory effects triggered by conventional radiotherapy (CRT) are highly dependent on single dose and radiation quality [5, 6]. Spatially Fractionated Radiation Therapy (SFRT) is another therapeutic approach with the potential to disrupt the classical paradigms of CRT [7–12]. In SFRT, the dose is spatially modulated to create highly non-uniform dose distributions, where oscillating regions of high dose, called peaks, and low dose, called valleys, fundamentally contrast with the flat dose profiles used in CRT [7–12]. The high spatial dose modulation in SFRT may activate distinct radiobiological mechanisms that minimize normal tissue toxicity while maintaining a significant therapeutic index, especially in bulk, radioresistant tumors [7–12]. Although the biological mechanisms underlying SFRT remain to be fully elucidated, preclinical studies indicate that its therapeutic effects may driven by distinct vascular alterations [13], inflammatory and immunomodulatory effects [14], the radiation-induced bystander effect (RIBE) [15], and the abscopal effect [7]. SFRT has been shown to induce immunogenic cell death (ICD) and promote the release of damage-associated molecular patterns (DAMPs) within high-dose regions, while concurrently preserving vascular integrity in low-dose regions [13, 16–20]. Preclinical models have demonstrated that SFRT can reshape the immunosuppressive tumor microenvironment (TME) by promoting the infiltration of cytotoxic T lymphocytes (CTLs) and natural killer (NK) cells, while concurrently depleting regulatory T cells (Tregs). Furthermore, SFRT can elicit systemic anti-tumor immunity at distant, unirradiated tumor sites, primarily mediated by the abscopal effect [21, 22]. Together, these multifaceted mechanisms culminate in a coordinated remodeling of the TME [23, 24]. Clinically, SFRT has been largely regarded as a cytoreductive approach for large-volume tumors, primarily used in palliative settings. Studies on the immune mechanisms of SFRT remain relatively limited, often restricted to preliminary experiments focusing on specific applications or patient populations. For this narrative review, we searched up to 2025 to retrieve original articles. Preclinical studies exploring the antitumor mechanisms of SFRT were identified from publications over the past approximately 20 years, since relevant mechanistic evidence has gradually accumulated within this research field. Although the concept of SFRT was proposed in the early 20th century, its clinical applications remained sporadic and supported by low-level evidence due to technical constraints. Advances in intensity-modulated RT, image guidance, and dosimetric calculation have facilitated the evolution of modern SFRT techniques such as LRT and MBRT. Accordingly, we limited inclusion to clinical studies published in the past decade to better reflect the contemporary evidence base and current clinical practice. All included preclinical and clinical studies were classified according to specific SFRT modality. Furthermore, preclinical studies were additionally grouped based on biological endpoints closely associated with antitumor immunity. This review aims to bridge the gap between these two perspectives. We first provide a critical assessment of the current clinical application of SFRT, highlighting its observed therapeutic efficacy and the limitations of these studies. We then discuss in depth the preclinical antitumor mechanisms, explore the potential of combining SFRT with immunotherapy to improve therapeutic outcomes, and identify key gaps between promising preclinical findings and clinical validation.

## Immune modulatory effects of RT

RT was long thought to be only a localized form of therapy, with a cascade of biological events, including the induction of DNA damage, the generation of reactive oxygen species (ROS), and the promotion of endothelial cell apoptosis [3, 4]. However, a paradigm shift has recently occurred in radiation oncology: RT can activate the host immune system and trigger systemic, immune-mediated effects, thereby enhancing the clearance of tumor cells [2]. Findings from preclinical and clinical studies consistently indicate that RT’s ability to elicit local and systemic antitumor immune activity is most pronounced when combined with immunotherapies that further modulate these responses [25, 26].

### Immune-activating effects of RT

Multiple RT modalities have been shown to elicit anti-tumor immune responses. First, preclinical investigations utilizing immunocompetent C57BL/6 mouse models of orthotopic ovarian cancer (ID8) and subcutaneous lung cancer (LLC) demonstrated that low-dose radiotherapy (LD-RT) reverses immune desertification within tumors, thereby enhancing responsiveness to immunotherapy [27]. Consistently, the capacity of LD-RT to remodel the immunosuppressive tumor stroma and potentiate systemic anti-tumor immunity has been demonstrated in bilateral 344SQ lung adenocarcinoma-bearing 129 Sv/Ev mice, characterized by enhanced M1 macrophage polarization, elevated NK cell infiltration, and reduced transforming growth factor-beta (TGF-β) levels [28]. Crucially, these preclinical mechanisms align with corroborating clinical data from human patients.

In addition, hypofractionated RT has also been shown to remodel the tumor immune microenvironment (TIME) through multiple mechanisms. Specifically, preclinical studies in both in vitro and in vivo settings have demonstrated that a single high-dose irradiation (e.g., 8 Gy (Gy)) upregulates the death receptor Fas, thereby enhancing tumor cell susceptibility to T cell-mediated killing via Fas/FasL-triggered apoptosis [29]. In immunocompetent BALB/c mice bearing A20 lymphoma (a syngeneic model), ablative radiation doses have also been shown to remodel the TIME by reducing the accumulation of immunosuppressive myeloid-derived suppressor cells (MDSCs) and promoting CTL infiltration into tumors [30]. Furthermore, in a B16 melanoma model using immunocompetent C57BL/6 mice, single high-dose radiation particularly enhances CTLs responses to major histocompatibility complex class I (MHC-I)-restricted antigenic peptides and augments the cytotoxicity of tumor-infiltrating lymphocytes (TILs) [31]. Notably, these immunostimulatory effects are not limited to single ultra-high doses: in a mouse melanoma model, a 2 × 7.5 Gy fractionation regimen provided better tumor control than a single 15 Gy dose and induced the stronger CTL response with the lowest Treg levels [32]. Meanwhile, it induced the strongest CTL response and maintained the lowest level of immunosuppressive Tregs [32]. Preclinical studies from multiple murine tumor models indicate that the immunostimulatory effects of RT are highly dependent on fractionation, dose, and scheduling, with optimal regimens differing across tumor types [33–35]. Thus, optimizing fractional doses and treatment intervals for distinct malignancies is essential to unleash the full potential of anti-tumor immunity.

### Immunosuppressive effects of RT

In addition to immune activation, RT has also been found to exert immunosuppressive effects. Radiation can simultaneously recruit immunosuppressive cells into the TME, thus weakening the overall immune response [36]. For instance, in a transgenic TRAMP-C1 prostate cancer model, delivery of a single high-dose fraction of 25 Gy to the tumor led to marked recruitment and activation of MDSCs within the tumor microenvironment [37]. MDSCs can establish an immunosuppressive microenvironment through multiple mechanisms, which results in the suppression of local immune responses [38]. Additionally, using the TRAMP-C1 prostate cancer model in C57BL/6 mice, it was demonstrated that RT can enhance Treg infiltration and accumulation in tumor tissues [39]. The core function of Tregs is to inhibit immune responses via various mechanisms [40]. In the immunocompetent C57BL/6 mouse model of AT3-OVA breast cancer, both 1 × 20 Gy and 3 × 4 Gy regimens resulted in enhanced local tumor control compared to RT without when Treg was depleted before RT [41]. However, the impact of RT on Tregs varies across different dosing regimens. A single high-dose fraction (e.g., 1 × 20 Gy) has been shown to rapidly induce profound Treg enrichment, which subsequently dampens the activation and effector function of CD8⁺ T cells, thereby compromising the synergistic efficacy between RT and anti-PD-1 immunotherapy [41]. This phenomenon, however, was not observed with low-to-moderate dose fractionation regimens (e.g., 3 × 4 Gy) [41]. Furthermore, Treg responses are tumor-type-dependent. For example, the same single high-dose RT regimen did not result in significant Treg enrichment in the MC38 colon cancer model, suggesting that the immunomodulatory effects of RT require tumor-specific adjustments based on TME characteristics [41]. Additionally, the direct physical damage caused by RT to the immune system is particularly prominent, manifesting as dose-dependent lymphopenia. Clinical data have clearly quantified this risk: in lung cancer patients receiving combined immunotherapy and RT, CRT (45 Gy in 15 fractions) induces significant lymphopenia (i.e., immunosuppression), and the severity of which is significantly correlated with lung V5 (the volume of lung tissue receiving ≥ 5 Gy) [42]. This treatment-related immune exhaustion has clear negative clinical implications. Among lung cancer patients treated with durvalumab following concurrent chemoradiotherapy, those with severe post-RT lymphopenia experience significantly reduced survival benefits from immune consolidation therapy [43].

Beyond the recruitment of immunosuppressive cells mentioned above, preclinical studies have demonstrated that RT, especially conventional and hypofractionated RT, may enhance the immunosuppressive TME by inducing programmed death ligand 1 (PD-L1) expression on tumor cell surfaces [44]. In addition, RT can also modify immunosuppressive cytokines. Ionizing radiation can directly activate latent TGF-β in the TME via inducing ROS, thereby converting it into a biologically active form [45]. Experiments using human breast cancer cell lines (MCF7, MDA‑MB‑231) demonstrated that pretreatment with a small-molecule TGF-β inhibitor significantly reduced clonogenic survival and increased their radiosensitivity [46]. Similarly, in vivo animal experiments demonstrated that pretreatment with a TGF-β neutralizing antibody (1D11) combined with RT significantly delayed tumor growth and reduced final tumor volume compared with either single-dose (8 Gy) or fractionated (3 × 12 Gy) RT alone [46]. In a separate study using a mouse breast cancer model with low immunogenicity, pretreatment with the TGF-β neutralizing antibody (1D11) before local RT, was found to increase the expression of activation markers (CD40/CD70) on intratumoral dendritic cells (DCs) induce CD8⁺ T cell responses against multiple endogenous tumor antigens (e.g., survivin, Twist, gp70), and achieve control of abscopal tumors compared to RT alone [47]. Moreover, combining this approach with anti-PD-1 therapy further improved tumor regression and prolonged survival. This indirectly indicates that TGF-β may inhibit anti-tumor immune responses by blocking the priming of CD8⁺ T cells against endogenous tumor antigens, thereby impairing the systemic immune effects triggered by RT [47].

In summary, uniform RT shapes the TIME through a dynamic process. The immune activation signals it induced (e.g., antigen release, CTLs priming) compete with immunosuppressive signals (e.g., MDSC/Treg recruitment, lymphopenia). Whether the outcome of TIME shaping leans toward immune activation or immunosuppression is likely determined not by a single mechanism but rather by the interaction between RT parameters (dose, fractionation) and the tumor’s specific immune context. This uncertainty and the inevitable immunosuppression, underscores the necessity of optimizing RT parameters to maximize the therapeutic synergy between CRT and immunotherapy [34]. Therefore, there is an urgent clinical need for an innovative RT modality that transcends the traditional “double-edged sword” paradigm. Ideally, this modality should spatially decouple the activating and suppressive effects of RT simultaneously, thereby shaping a TME dominated by immune activation in a more stable and controllable manner.

It is this goal that has made SFRT a research focus, owing to its unique physical dose-distribution characteristics. Unlike uniform irradiation of the entire target volume, SFRT enables the simultaneous delivery of spatially alternating, extremely high peak doses and very low valley doses within the tumor during a single treatment session [23]. Below, we will elaborate on the types of SFRT, its clinical applications, how SFRT shapes a stable TIME dominated by immune activation, and how the combination of SFRT and immunotherapy achieves optimal anti-tumor immunity.

## Novel SFRT techniques

**Fig. 1:**
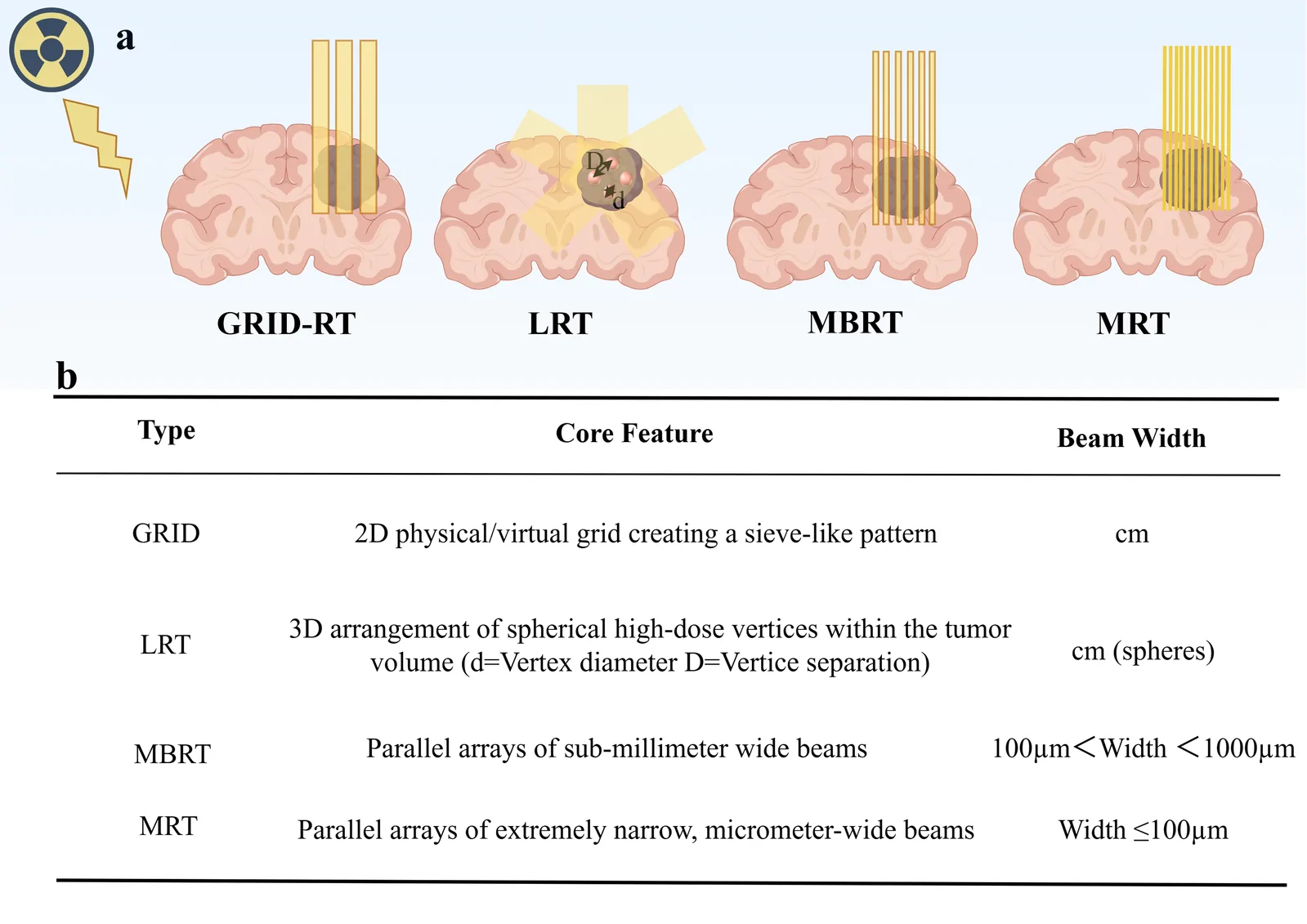
Schematic comparison of the four primary SFRT techniques. (**a**) Conceptual diagram depicting the fundamental principle of SFRT, where a highly heterogeneous dose distribution is intentionally created within the target volume. (**b**) The table below systematically compares the core features and beam width parameters of the four primary SFRT techniques: GRID-RT, characterized by the use of broad, centimeter (cm)-wide beams to create a two-dimensional dose pattern; LRT, defined by a three-dimensional arrangement of high-dose spherical vertices within the tumor. The geometry is specified by the vertex diameter and the center-to-center vertex separation. MBRT, characterized by narrow, sub-millimeter beams ranging from 100 to 1000 μm in width. MRT: defined by the narrowest beam arrays with widths of 100 μm or less to generate the most extreme dose gradients

The novel SFRT model achieves a breakthrough over conventional uniform-dose RT by spatially alternating high-dose (“peak”) and low-dose (“valley”) regions, enabling a single high-dose radiation delivery [23]. SFRT techniques primarily include GRID RT, lattice radiotherapy (LRT), mini-beam radiotherapy (MBRT), and microbeam radiotherapy (MRT). A schematic comparison of these four SFRT techniques is illustrated in Fig. 1. Although these techniques vary in execution, they all share the common goal of generating a spatially heterogeneous dose distribution within the target tumor [9, 48–50].

Traditional two-dimensional (2D) GRID RT employs a physical grid block to segment a single high-dose radiation beam into numerous small beamlets, generating a “checkerboard” pattern of high peak doses and low valley doses at the centimeter scale within the target volume [51]. However, modern treatment planning systems can achieve more precise three-dimensional (3D) dose sculpting using multi-leaf collimators (MLCs). LRT represents the three-dimensional evolution of GRID RT within the context of modern radiation therapy technologies [52]. It works by creating multiple dispersed, spatially arranged high-dose vertices within the tumor volume, which are surrounded by lower-dose regions [52]. Compared to GRID RT, LRT can achieve superior dose conformity. The high-dose regions can more precisely target radioresistant sub-volumes within the tumor while better sparing sensitive normal structures interspersed inside the tumor, such as blood vessels and nerves [49]. At a finer scale, MBRT utilizes sub-millimeter beams and steep dose gradients. This approach enhances tumor dose delivery while maintaining high tolerance in normal tissues, thereby improving the therapeutic ratio [9, 14, 53, 54]. Finally, MRT employs even narrower, parallel arrays of beams with micrometer (µm)-scale widths. Leveraging its extremely high dose rate and low beam divergence, MRT can deliver peak doses of several hundred Gy to the target [16, 50, 55].

Table 1 summarizes a representative selection of preclinical and clinical studies that have shaped the current understanding of SFRT. Preclinical studies provided insights into key mechanisms underlying SFRT-mediated anti-tumor effects. In contrast, clinical studies have largely reported efficacy and safety outcomes across various tumor types, with some offering indirect insights into its potential immunomodulatory role. It can be observed that the clinical application of SFRT (except for MRT, which remains in preclinical research) is primarily focused on palliative cytoreduction of large-volume tumors. In certain reported cases, it has been combined with CRT for drug-resistant patients with limited life expectancy who have undergone multiple lines of treatment. Although encouraging tumor regression has been reported in isolated case reports, the overall clinical evidence is largely based on small-sample retrospective studies and case reports, lacking prospective controlled trials and systematic immune monitoring. Therefore, most studies investigating the mechanisms of action of SFRT have focused on preclinical models, and how various forms of SFRT reshape the TME are being gradually explored. Despite technical differences, GRID RT, LRT, MBRT, and MRT may reshape the immunosuppressive TME and could contribute to anti-tumor immune responses through their unique spatial dose geometries. However, the strength of evidence varies across modalities. In addition, the table also introduces partial irradiation (pIR), a simple form of spatial fractionation.

**Table 1:** Key findings from preclinical and clinical studies on SFRT

Ref.	Model/Tumor	Tumor Volume	SFRT Type	Key Findings	Study Design	Immune Monitoring	Clinical Status
[95]	Balb/c (EMT6.5)	NA	MRT	Several immune-related genes showed differential expression (vs. CRT)	NA	NA	Preclinic
[119]	C57BL/6(4TI)	NA	MRT	Reduction in tumor vascular density; increased pericyte coverage; high-dose MRT leading to decreased tumor hypoxia	NA	NA	Preclinic
[83]	F344 (9 L)	NA	MRT	Early changes in immune signaling pathways in both the tumor and the normal brain	NA	NA	Preclinic
[7]	C57BL/6 (LLC1)	NA	LRT	Irradiation at 20% volume results in delayed abscopal tumor growth, increased intratumoral CD3⁺ T cells, and higher serum IFN‑γ levels	NA	NA	Preclinic
[123]	nude mice(4T1)	NA	MRT	Tumor and normal tissue microvascular dynamics differed from those during CRT; HIF‑1α exhibited spatial and temporal changes	NA	NA	Preclinic
[84]	F344 (9 L)	NA	MRT	Longer survival (vs. CRT); increased cell death and macrophage recruitment	NA	NA	Preclinic
[117]	F344 (F98)	NA	MRT	Tumor vascular permeability increased from day 2 to 14 (peak + 44.8% at day 7); CRT showed an increase only at day 7 (+ 19.1%); normal brain vessels remained unchanged	NA	NA	Preclinic
[94]	C57BL/6	NA	MRT	Systemic oxidative DNA damage and inflammation in distant tissues; increased macrophages, neutrophils, and T cells; altered of serum cytokines	NA	NA	Preclinic
[144]	C57BL/6	NA	MRT	No systemic γH2AX in NSG or CCL2‑KO mice; present in wild‑type	NA	NA	Preclinic
[85]	C57BL/6 (B16-F10)	NA	MRT	Tumor growth was slower (vs. CRT), with increased intratumoral CD8⁺ T cells, NK cells, macrophages, senescent cells, MCP-1, and MIP-1α	NA	NA	Preclinic
[86]	Balb/c (EMT6.5)	NA	MRT	Reduction in immunosuppressive myeloid cells; increase in cytotoxic T cells (vs. CRT)	NA	NA	Preclinic
[81]	Balb/c (67NR); C57BL/6 (LLC); nude mice	NA	pIR	Tumor growth delay with pIR equal to CRT in immunocompetent mice; no delay in nude mice	NA	NA	Preclinic
[82]	C57BL/6	NA	MRT	Broader and more sustained changes in immune‑ and inflammation‑related gene expression occurred in the brainstem (vs. CRT)	NA	NA	Preclinic
[21]	C57BL/6 (B16-F10)	NA	MBRT	CD8⁺ T cell-dependent tumor control; increased intratumoral CD8⁺ T cells and B cells; no change in Tregs; upregulation of CXCL9	NA	NA	Preclinic
[14]	F344 (RG2)	NA	MBRT	Prolonged survival; increase in tumor‑infiltrating CD8⁺/CD4⁺ T cells in tumor core; long‑term antitumor immunity; lower normal brain toxicity (vs. CRT)	NA	NA	Preclinic
[80]	Rat (RG2)	NA	MBRT	Higher PVDR was associated with reduced infiltration of Tregs and pro‑inflammatory myeloid cells and with better tumor control	NA	NA	Preclinic
[22]	C57BL/6 (MC38); BALB/c (CT26, 4T1)	NA	pIR	Infiltration of CD8 + T cells and NK cells; increased expression of CCL5, CXCL1, and CXCL2; a shift in CD8 + T cells toward an IFN-activated cytotoxic phenotype	NA	NA	Preclinic
[99]	F344 (RG2)	NA	MBRT	Prolonged survival; increased CD8⁺ T cells, B cells, and DCs; promotion of T cell infiltration into the tumor core	NA	NA	Preclinic
[147]	C57BL/6 (B16-F10)	NA	MRT	Increase in CD8⁺ T cell infiltration and antigen presentation; loss of efficacy after CD8⁺ T cell depletion; reduction in locoregional metastasis	NA	NA	Preclinic
[162]	Ovarian carcinosarcoma	GTV 1495 cm³	LRT + CFRT	> 70% volume reduction with > 4‑year local control; patient survived 50 months	Case report	No	Clinic
[163]	Bulky NSCLC	Mean GTV 195 cm³	LRT + CFRT	42% mean tumor reduction; 16‑month median OS; no LRT‑related mortality or significant toxicity	Retrospective case series	No	Clinic
[56]	Bulky H&N tumors	Median 9.5 cm	GRID + CFRT	54.5% experienced palliative symptom improvement; 44.4% achieved CR in the definitive group; grade ≥ 3 skin toxicity occurred in 23.8%	Retrospective case series	No	Clinic
[63]	Far-advanced bulky cervical cancer	Mean GTV 200 cm³	LRT + CFRT	100% local control, 88.9% CMR, 53.3% 2‑year DSS; no grade ≥ 3 toxicity	Retrospective case series	No	Clinic
[58]	Various bulky refractory tumors	Mean 649 cm³	GRID + ART	A melanoma patient resistant to ICIs was re‑sensitized	Case report	No	Clinic
[60]	Soft tissue sarcoma, osteosarcoma	Median 14.2 cm	GRID + CFRT	35.3% pCR in high‑grade sarcomas (exceeding 19.4% historical control); 2‑year LRC 85%	Retrospective case series	No	Clinic
[52]	Various bulky tumors	Median GTV 146.8 cm³	LRT+ HFRT	100% symptomatic response, 89% overall clinical response (23% CR); 1‑year OS 86.4%; mild acute toxicity in 20%	Retrospective multicenter series	No	Clinic
[62]	Uterine serous papillary carcinoma	GTV 542.8 cm³	LRT + CFRT	Near‑complete tumor regression and 20‑month survival without toxicity; MRI‑based approach effective and safe	Case report	No	Clinic
[64]	Various large tumors	Median GTV 415 cm³	LRT	87% rapid symptom relief; 80% lesion shrinkage (mean 48%); mean response duration of 7.4 months	Retrospective case series	No	Clinic
[70]	Merkel cell carcinoma(pt1); Recurrent SCC (pt2)	14 × 14 × 11 cm³; 7 × 6 × 3 cm³	MBRT	First-in-human MBRT: relief and tumor response (63% volume reduction at 3 weeks in pt1; complete flattening at 34 days in pt2); minimal toxicity	Case report	No	Clinic
[65]	Large extracranial solid cancers	Median 560 cm³	LRT	79% 3‑month ORR with 54% median tumor shrinkage; all patients had rapid pain relief; no grade ≥ 3 toxicity	Retrospective case series	No	Clinic
[71]	Recurrent mucosal melanoma	4.1 cm	MBRT	Complete metabolic and clinical response with abscopal effect in untreated metastasis; minimal toxicity > 6 months	Case report	No	Clinic
[164]	High‑grade extremity spindle cell sarcoma	GTV926 cm³	GRID + EBRT	Tumor regression 90% and necrosis 99% were observed.	Case report	No	Clinic
[67]	Soft tissue and bone sarcomas	Median GTV 636 cm³	LRT + EBRT	Symptom relief was documented in 60% of treatment courses; stable or partial response was observed in 90%; 1-year overall survival was 53%, and local control was 82%	Retrospective case series	No	Clinic
[68]	Soft tissue sarcomas	Median GTV 1058 cm³	LRT + EBRT	All symptomatic patients reported symptom relief. At 1–2 months, 67% had stable disease. Surgical resection became feasible in 5/15 patients; 3 remained alive without disease	Retrospective case series	No	Clinic
[69]	Various large tumors	Median GTV 415 cm³	LRT	Rapid shrinkage (75% of lesions) and lifelong symptom relief	Retrospective case series	No	Clinic

Abbreviations: CFRT = conventionally fractionated radiotherapy; HFRT = hypofractionated radiotherapy; ART = adjuvant radiotherapy; γH2AX = phosphorylated histone H2AX; NSG = NOD scid gamma; CCL2‑KO = CCL2 knockout; GTV = gross tumor volume; H&N = head and neck; CMR = complete metabolic response; DSS = disease-specific survival; OS = overall survival; MRI = magnetic resonance imaging; SCC = squamous cell carcinoma; pt = patient; ORR = objective response rate; NA = not available. Preclinical studies are listed in chronological order; clinical studies are from the past decade

## Clinical applications of SFRT

### GRID

As the earliest clinically investigated SFRT modality, GRID RT has provided foundational evidence linking the physical dose distribution to systemic immune activation. A retrospective analysis of 21 patients with bulky head and neck tumors (median 9.5 cm) treated with GRID RT followed by CRT reported that 44.4% of curative-intent patients receiving concurrent chemotherapy achieved complete response (CR) [56]. More importantly, Sathishkumar et al. [8] provided the clinical evidence that GRID RT induces systemic immune activation: serum tumor necrosis factor-alpha (TNF‑α) levels were significantly elevated after GRID RT treatment, which correlated with complete tumor regression. As a key proinflammatory cytokine, TNF‑α promotes DCs maturation and T‑cell activation, indicating that GRID RT may convert immunotherapy‑resistant “cold” tumors into “hot” tumors [57]. Two subsequent case reports further support this immunomodulatory potential: proton GRID RT re-sensitized a pembrolizumab-resistant melanoma patient to immunotherapy [58], and single-fraction GRID RT combined with pembrolizumab induced complete regression of a bulky melanoma mass (18 × 15 × 8 cm) in a patient with dual resistance to ipilimumab and pembrolizumab [59].

Nevertheless, most clinical reports still focus on the tumor control and normal tissue protection of SFRT. In the neoadjuvant setting, GRID RT followed by CRT and surgery was administered to 26 patients with bulky high-risk sarcomas (median size 14.2 cm), resulting in a significantly improved pathologic complete response (pCR) [60]. Another preoperative dosimetric study in breast cancer explored the potential of photon mini‑GRID RT. This technique uses microbeams of 1.8 mm width with 3.5 mm spacing, delivering a single dose of approximately 15 Gy [61]. It reduced the mean doses to the lung, heart, chest wall, and lymph nodes by 50%-100%, and the skin dose by 20%-60%. Furthermore, the doses to all organs at risk were within the tolerance limits specified in TG‑101 report of the American Association of Physicists in Medicine (AAPM) (dose constraints for normal tissues in stereotactic body radiation therapy) [61].

Despite these encouraging observations, several methodological limitations temper their interpretability. Most GRID RT studies are small retrospective case series with heterogeneous patient cohorts [56, 60] and lack control groups and prospective immune monitoring. Although TNF-α elevation correlated with response [8], no study dynamically tracked tumor-infiltrating lymphocytes or changes in the broader immune profile, making it impossible to directly attribute this single-cytokine increase to clinically meaningful adaptive immune responses. The cross-trial comparison of pCR rates in sarcomas [60] is confounded by differences in patient selection, chemotherapy regimens, and surgical timing. Thus, while GRID RT shows promise as an immune primer, head-to-head prospective comparisons with standard therapy are urgently needed.

### LRT

LRT has generated substantial clinical data across multiple tumor types, evolving from feasibility studies to functional imaging-guided approaches. Metabolism-guided LRT in 30 patients with bulky stage IV tumors achieved faster symptom relief than conventional palliative RT, with 100% symptom response and 89% clinical response rates [52]. Parallel efforts using MRI-guided LRT in recurrent uterine cancer produced 87.4% tumor shrinkage and 20-month progression-free survival without chemotherapy [62]. In cervical cancer, LRT with concurrent cisplatin in 10 patients achieved 100% local control and an 88.9% complete metabolic response. However, there was a striking histologic disparity: the 2-year disease-specific survival rate was 100% for squamous cell carcinoma, but 0% for adenocarcinoma/adenosquamous carcinoma (*P* = 0.01) [63]. Larger retrospective series have since validated these findings across diverse tumors. Studer et al. [64]. reported 80% radiographic response in 56 bulky lesions (median 415 cc), noting significant histologic variation among sarcomas, carcinomas, and melanomas. Similarly, Iori et al. [65]. used a 5-fraction regimen in 20 patients, achieving 79% objective response at 3 months and universal rapid pain relief. Complementing these efficacy data, a Phase I trial involving 20 patients with large tumors (> 5 cm) reported no acute toxicities of grade ≥ 3, reinforcing the favorable safety profile of LRT [66]. Collectively, these findings demonstrate that LRT has accumulated substantial clinical data across multiple tumor types, with reported efficacy, ability to integrate functional imaging, and a favorable safety profile, characteristics that support further investigation of LRT as a potential platform for future combination strategies.

Recent sarcoma-focused studies have further nuanced this picture. Ahmed et al. [67]. reported the largest SFRT sarcoma series to date (53 patients, 85% volumetric modulated arc therapy (VMAT) Lattice), showing 60% symptom relief and 90% radiographic response when combined with subsequent external beam radiotherapy (EBRT). However, grade 3–4 toxicities occurred in 4 patients (8%), including one intestinal fistula directly attributed to peak placement adjacent to bowel, underscoring the critical importance of treatment planning and the potential hazards of early clinical translation [67]. Another study of unresectable non-extremity soft tissue sarcomas (*n* = 15) reported that LRT (20 Gy single fraction to peaks) with EBRT achieved 100% symptom relief and rendered 5 patients (33%) eligible for resection. However, pathologic response rates varied widely (10–90%), and only 3 achieved disease-free survival [68]. A recent comparative study enrolled 66 patients with 81 lesions. The results showed that single-fraction stereotactic body radiotherapy (SBRT)-LRT and 5-fraction simultaneous integrated boost (SIB)-LRT achieved comparable tumor reduction of approximately 75% in carcinomas, sarcomas and melanomas. These findings challenge the simplistic concept of histology-determined efficacy proposed in cervical cancer studies [63], and suggest LRT may activate common, non-specific immune mechanisms.

These LRT studies collectively validate clinical feasibility and efficacy but reveal critical contradictions and gaps. While multiple studies invoke “immunomodulation” to explain efficacy [52, 62, 63], none direct immunological evidence was provided, such as TIL analysis or cytokine profiling. The study in cervical cancer found that the effectiveness of LRT varied by histologic type (squamous vs. adenocarcinoma) [63]. However, another larger study [69] reported similar effectiveness across different histologic types [69]. These conflicting findings raise a critical, unresolved question: Is the efficacy of LRT dependent on histology? Do tumors of different histological types harbor heterogeneous tumor immune microenvironments? The underlying mechanisms remain to be elucidated. Additionally, most studies use single-arm retrospective designs and lack head-to-head comparisons with standard therapy, hindering objective assessment of LRT’s true clinical value. Moreover, LRT has not been used as monotherapy, and its application has been restricted to the palliation of bulky tumors; therefore, its indications and combination regimens remain to be further explored.

### MBRT

In recent years, MBRT has achieved pivotal milestones in clinical translation. Grams et al. reported the clinical application of MBRT in two patients (one with chest wall sarcoma and the other with cervical squamous cell carcinoma) [70]. Concurrently, in vivo film dosimetry verified a distinct peak-to-valley dose separation [70]. Subsequently, Jacobson et al. described application of the MBRT in a patient with refractory mucosal melanoma after several lines of unsuccessful therapy (dual immune checkpoint inhibitors, chemotherapy, targeted therapy) [71]. It achieved a complete metabolic response at 13 weeks, with concomitant regression of an unirradiated abdominal metastasis, suggesting an abscopal effect [71]. Circulating tumor DNA became undetectable at 4 months, with toxicities limited to transient keratoconjunctivitis and grade 1 erythema [71]. An ongoing phase I trial (NCT07062003) is actively recruiting to determine the maximum tolerated dose of MBRT in up to 60 patients with recurrent or metastatic skin/soft tissue tumors.

Along this continuum of clinical translation, MRT has advanced toward human application through foundational physical dosimetry studies and compelling large-animal data. A dosimetric simulation study of MRT treatment planning [72], based on real-world data from five patients with different tumor types, introduced the concept of equivalent uniform dose (EUD) to compare dose distributions between MRT and CRT. The results confirmed that MRT can meet clinical dose constraints in most cases, providing a critical dosimetric foundation for its clinical application [72]. In a veterinary clinical trial of MRT for spontaneous gliomas in dogs [73], tumors exhibited an 87.4% reduction in volume at 3 months, seizures were completely controlled, and no toxicities were observed—representing a key milestone toward human clinical trials.

While these MBRT/MRT findings provide compelling proof of concept, their limitations must be acknowledged. This remarkable first human MBRT case remains a single, isolated report, and its generalizability has yet to be demonstrated [71]. The patient received MBRT followed by TIL therapy. Although a radiological CR occurred before TIL infusion, the contributory role of subsequent TIL therapy cannot be entirely excluded. Whether MBRT-induced immune activation confers durable immune memory or merely creates a favorable “priming” microenvironment for subsequent therapy remains unknown. The veterinary glioma trial [73], while important, represents a single case with only a 3-month follow-up; long-term safety and efficacy in humans await investigation.

Based on these clinical studies of SFRT, although the evidence base remains dominated by small retrospective series and case reports, growing clinical evidence implies that increased serum TNF‑α levels post GRID RT may correlate with abscopal effect manifestation [71], as well as the reversal of established resistance to immunotherapy after GRID RT [57–59]. Given their origin in small, uncontrolled cohorts, these findings remain hypothesis-generating rather than conclusive. Such studies are inherently susceptible to numerous confounders, including concurrent systemic therapies, patient selection bias, and the lack of standardized immunomonitoring frameworks. Accordingly, these results cannot be regarded as definitive evidence of therapeutic efficacy. Well-designed prospective controlled trials with predefined immune correlative endpoints are therefore urgently warranted. Nevertheless, these clinical observations suggest that MBRT-induced antitumor effects may be closely associated with its underlying immunomodulatory functions. To date, in-depth investigations into the antitumor immune mechanisms of SFRT still rely predominantly on preclinical studies. Under controlled experimental conditions, these studies can provide insights into how SFRT reshapes the TIME and elicits specific antitumor immune responses. However, the generalizability of these findings across different SFRT techniques and tumor models remains to be determined. Therefore, in the next section, we will review preclinical evidence to discuss the proposed mechanisms underlying antitumor immunity induced by specific SFRT platforms, which may help inform future research rather than serve as a definitive theoretical foundation for clinical optimization.

## SFRT-mediated anti-tumor effects

**Fig. 2:**
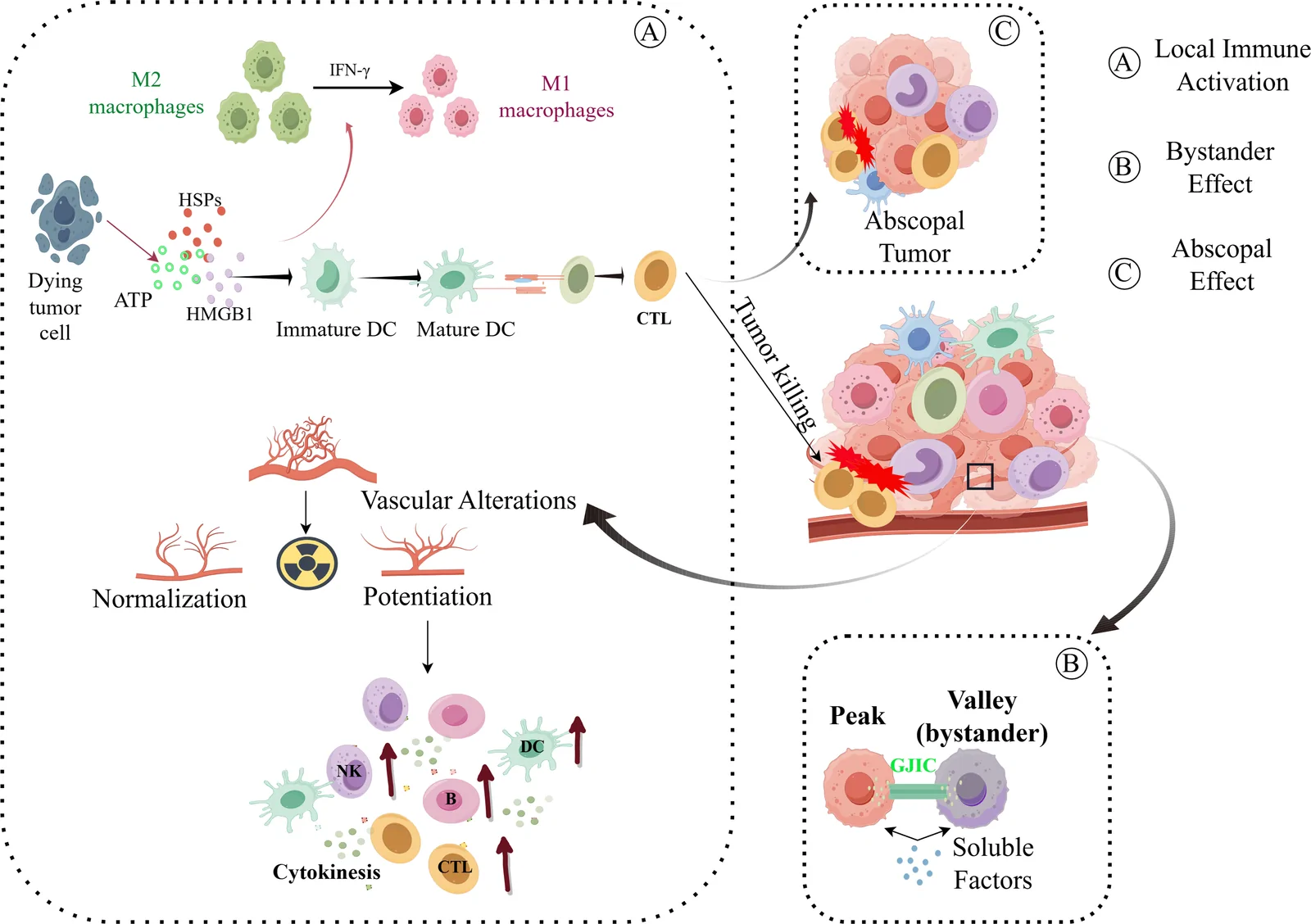
Schematic diagram illustrating the proposed immunomodulatory mechanisms of SFRT. (**A**) Local immune activation: SFRT induces ICD, releasing TAAs and DAMPs, such as ATP, HMGB1, and HSPs. This process promotes the DCs and enhances antigen presentation, thereby improving the recognition and killing capacity of CTLs. Meanwhile, the potentiated vascular facilitates the infiltration of effector immune cells, including CTLs, NK cells, DCs, and B cells, into the TME. Concurrently, activated immune cells release pro-inflammatory cytokines. Radiation also helps repolarize TAMs toward the pro-inflammatory M1 phenotype. (**B**) Regional immune extension via the radiation-induced bystander-like effect: soluble factors from irradiated cells trigger cytotoxic effects and immune regulation in low-dose bystander cells. (**C**) Systemic immune control through the abscopal effect: local immune activation initiates a systemic anti-tumor response. Activated CTLs circulate and infiltrate distant non-irradiated metastases, resulting in tumor regression

Based on existing research, SFRT is proposed to modulate the anti-tumor immune response through three interconnected mechanisms operating at distinct spatial scales: local immune activation within heterogeneous dose regions, regional cytotoxicity mediated by the RIBE, and systemic disease control via the abscopal effect (Fig. 2).

### Local immune activation

#### Immunogenic cell death (ICD)

Within the high-dose regions, SFRT causes direct physical destruction, by inducing DNA double-strand breaks and apoptosis of tumor cells, which creates a spatially confined DNA damage [19, 74]. For example, when a reconstructed human epidermis (epiCS) model was irradiated with planar proton microbeams of different widths, focused 66 μm microbeams induced highly localized clusters of γ‑H2AX–positive cells strictly along the beam path, whereas the tissue between the beams remained completely unaffected [74]. Although this elegantly demonstrated the spatial confinement of DNA damage, the in vitro skin equivalent lacks a functional immune system. It therefore does not permit assessment of immune cell recruitment, antigen presentation, or T-cell priming, which is the key processes underlying SFRT’s proposed immunomodulatory effects. In an intact functional immune system, this severe DNA damage at high-dose peaks can induce ICD and release large amounts of tumor-associated antigens (TAAs) and DAMPs such as adenosine triphosphate (ATP), high mobility group box 1 (HMGB1), and heat shock proteins (HSPs), thereby enhancing the antigen-presenting capacity of DCs [23, 24, 75]. The released DAMPs can be recognized by receptors on macrophages, promoting their polarization toward a pro-inflammatory M1 phenotype [76]. Notably, the high single doses delivered to these sub-volumes (often > 12–18 Gy) can, in parallel, induce the expression of the DNA exonuclease three prime repair exonuclease 1 (Trex1), which degrades cytosolic DNA and thereby limits the activation of the cyclic GMP-AMP synthase-stimulator of interferon genes (cGAS-STING) pathway and subsequent interferon-beta (IFN-β) production within these same regions [77]. In contrast, adjacent low-dose radiation does not exhibit Trex1 upregulation. This allows for the accumulation of cytosolic double-stranded DNA, robust activation of the cGAS-STING pathway, and enhanced secretion of IFN-β—processes that are critical for the recruitment of DCs and the priming of CD8⁺ T cells [77]. Thus, it has been hypothesized that low-dose irradiation may critically counteract the Trex1-mediated immunosuppressive effect in high-dose spots and reprogram the immunosuppressive TME toward a more immunogenic state [27, 78]. However, this hypothesis is extrapolated from CRT studies and has not been directly validated in the context of SFRT. Although the Trex1-cGAS-STING axis serves as a dose-dependent switch determining immunogenicity in CRT, its activation threshold, spatial expression pattern, and net contribution to anti-tumor immunity remain completely unexplored in SFRT [77]. This knowledge gap is directly tied to the unique spatial complexity introduced by the coexistence of high-dose peaks and low-dose valleys within a single tumor.

In another study using MBRT in Fischer 344 rats bearing fibrosarcoma, the results showed that the valley dose, rather than the peak dose, was most strongly correlated with tumor response [79]. This interesting finding suggests that, in addition to ICD induced by high-dose peaks [23, 24, 75], the valley dose regions exhibit unique biological responses that critically influence treatment outcomes. Subsequent studies integrating both peak and valley doses revealed that greater dose heterogeneity, achieved by optimizing peak doses while maintaining minimal valley doses, can promote the formation of a less immunosuppressive TME [80]. This underscores the critical need to further elucidate the spatial and temporal crosstalk between immune activation and suppression within peak‑dose and valley‑dose regions during SFRT, and to establish optimized dose‑distribution principles that maximize synergistic anti‑tumor immunity for future clinical translation.

#### Immune cell recruitment and activation with cytokine network modulation

Immune cells infiltrating the TME are the core mediators of the anti-tumor immune effects exerted by SFRT, and complex cytokine and chemokine networks orchestrate their recruitment and function. Specifically, using 67NR orthotopic breast tumors and subcutaneous LLC tumors in immunocompetent mice models, it demonstrated that pIR (partial irradiation, a type of SFRT) achieved outcomes comparable to whole-tumor irradiation in terms of tumor growth delay and cure rate, suggesting that pIR can attain the equivalent therapeutic efficacy of CRT [81]. Subsequent experiments further confirmed that the anti-tumor immune response induced by pIR is highly likely dependent on the immunomodulatory function of T cells [81].

Beyond T cells, the regulatory effects on other immune cell subsets have been progressively elucidated in preclinical models. Taking MRT as an example, Smyth et al. [82]. observed more persistent immune responses in the brainstem of healthy mice following MRT than broad-beam RT. In parallel, experimental studies employing orthotopic 9 L gliosarcoma‑bearing F344 rats have demonstrated that MRT can activate the related signaling pathways of multiple immune cell types, including macrophages, DCs, NK cells, and B cells [83, 84]. However, this study was based solely on transcriptomic data from a single early time point (6 h), lacking dynamic time‑course analysis and protein‑level validation. Consistent with these observations, comparative studies across multiple tumor models have further reported the superior immunomodulatory properties of MRT over CRT, with these advantages closely linked to differential regulation of cytokine networks. For example, in a radioresistant melanoma model, MRT not only achieved a more pronounced delay in tumor growth than conventional broad-beam RT but also induced tumor cell senescence and upregulated chemokines such as monocyte chemoattractant protein‑1 (MCP-1) and macrophage inflammatory protein-1 alpha (MIP-1α), thereby promoting the infiltration of CD8⁺ T cells, NK cells, and macrophages [85]. In contrast, the role of CRT in immune cell recruitment and systemic immune activation is notably weaker [85]. In immunocompetent C57BL/6 mice with EMT6.5 breast tumor, the discrepancy in immunomodulatory patterns between the two modalities is even more fundamental: CRT markedly increases the infiltration of tumor-associated macrophages (TAMs), neutrophils, and the expression of C-C motif chemokine ligand 2 (CCL2), where these cells and factors are mostly involved in immunosuppression. By contrast, MRT avoids the accumulation of such immunosuppressive cells, instead promoting CD3⁺ T cell infiltration and inducing higher levels of pro-inflammatory gene expression, ultimately shaping a TME more conducive to anti-tumor immunity [86]. Consistent with these in vivo observations, in vitro studies using EMT6.5 mouse breast cancer cells have shown that MRT modulates the expression of chemokines, including CCL2, C-C motif chemokine ligand 5 (CCL5), and granulocyte-macrophage colony-stimulating factor (GM-CSF) [87]. Studies have clarified the functional roles of these molecules: CCL2 primarily mediates the migration of monocytes and macrophages, CCL5 recruits T cells and NK cells, and GM-CSF enhances antigen presentation by promoting DCs maturation [88–93]. Together, they form a synergistic regulatory network that drives the accumulation of effector immune cells in the tumor region [88–93]. Thus, both in vivo and in vitro evidence are consistent with the hypothesis that MRT orchestrates a distinct immunomodulatory profile characterized by reduced immunosuppressive signaling and enhanced recruitment of effector immune populations. However, these findings are platform‑ and model‑dependent; generalizing them to all SFRT modalities or to human patients is not yet justified.

Further supporting the immune-activating potential of MRT, localized MRT in C57BL/6 mouse models, has been shown to trigger systemic responses, including oxidative DNA damage and immune cell infiltration (macrophages, neutrophils, T cells) in distant normal tissues [94]. In addition, MRT caused clear changes in cytokine levels. For example, it decreased eotaxin, macrophage-derived chemokine (MDC), and interleukin-10 (IL-10). At the same time, it increased tissue inhibitor of metalloproteinases 1 (TIMP1), vascular endothelial growth factor (VEGF), and transforming growth factor-β (TGF-β) [94]. Although these studies were performed in normal tissues, the findings suggest that MRT can induce immunomodulation that is not restricted to the irradiated field, but involves coordinated local and systemic interactions between immune cells and cytokines.

Despite these promising findings, critical methodological considerations and knowledge gaps remain. Studies using the same MRT technique in different experimental systems have yielded divergent results. For instance, in mouse breast cancer models, upregulation of C-X-C motif chemokine ligand 1 (CXCL1), a key chemokine for recruiting MDSCs, and downregulation of C-X-C motif chemokine ligand 9 (CXCL9), a Th1-type chemokine critical for effector T cell recruitment, were observed [95]. Notably, this finding contrasts with the pro-inflammatory gene expression patterns reported in vitro. This discrepancy between in vitro and in vivo findings underscores a crucial point: results obtained from simplified in vitro systems may not reliably predict responses in the complex TME [87].

Temporal dynamics add another layer of complexity. In the same in vivo breast cancer model [95], unlike the 4-hour time point, numerous immunomodulatory genes were expressed at 24 and 48 h post-MRT. This indicates that MRT-induced biological effects, particularly those related to immunity, require time to establish. MRT induces a dynamic, nuanced, and specific immunomodulatory profile [87, 95]. Importantly, the observed transcriptional differences between MRT and CRT do not necessarily translate into differences in immune cell infiltration numbers; they may instead manifest at more sophisticated levels, such as cell activation status, functional polarization, or degranulation activity [96]. These findings demonstrate that MRT-induced immune modulation extends beyond the irradiated field and involves coordinated interactions between immune cells and cytokines at both local and systemic levels. Therefore, future studies must validate in vitro findings in appropriate in vivo models, incorporate multi-timepoint dynamic observations and protein-level validation, and employ techniques such as flow cytometry to truly understand MRT’s modulation of the entire TIME.

Notably, the immunoregulatory advantages of SFRT are not exclusive to MRT. LRT employing partial-volume, multi-vertex irradiation has also exhibited unique value in immunocompetent C57BL/6 mice with a bilateral LLC lung cancer. LRT treatment can simultaneously enhance CD3⁺ T cell infiltration in both irradiated and non-irradiated tumor lesions. In contrast, CRT only increases CD3⁺ T cell infiltration in irradiated tumors and even reduces CD3⁺ T cell infiltration in non-irradiated contralateral tumors [7]. In mice bearing bilateral LLC tumors, LRT (especially irradiation delivered at two 10% and 50% tumor vertices) induced a more robust Th1-type immune response compared with CRT, characterized by elevated interferon-gamma (IFN-γ) levels in the serum, increased secretion of tumor-specific IFN-γ and interleukin-2 (IL-2) by splenocytes, as well as significant suppression of the expression of interleukin-4 (IL-4) and IL-10, the two key Th2-type immunosuppressive cytokines [7]. This difference indicates that LRT can more effectively elicit systemic anti-tumor immune responses through its distinctive spatial dose distribution [7]. Similarly, in an immunocompetent mouse model of 4T1 triple-negative breast cancer, comparative studies between GRID RT and CRT have shown that GRID RT significantly delays the growth of abscopal tumors [97]. The underlying mechanism is closely associated with increased infiltration of activated CD8⁺/CD4⁺ T cells and DCs in irradiated tumors and abscopal tumors, elevated IFN-γ levels in peripheral blood, and upregulated PD-L1 expression in abscopal lesions [97]. This systemic upregulation of PD-L1 renders distant metastases uniquely vulnerable to subsequent anti-PD-1/PD-L1 checkpoint inhibition. Consequently, elevated PD-L1 expression at abscopal sites serves as both a surrogate biomarker of systemic immune activation and a strong mechanistic rationale for combining GRID RT with ICIs [98].

In addition, in orthotopic RG2 glioma‑bearing Fischer 344 rats, compared with conventional proton therapy delivered at the same average dose, proton MBRT significantly increased tumor‑infiltrating CD8⁺ tissue‑resident memory T cells, B cells, and conventional type 1 DCs, while uniquely promoting T cell infiltration into the tumor core [99]. These cellular changes are beneficial for anti-tumor immunity. Tissue-resident memory CD8⁺ T cells can remain within the tumor and act as sentinels that help prevent tumor recurrence [100]. In addition, increased infiltration of B cells and DCs suggests that the immune system is more effectively activated to recognize and eliminate tumor cells, a process known as the “priming” of adaptive immunity [101]. This pattern suggests that MBRT may enhance both the priming and the long-term surveillance phases of anti-tumor immunity more effectively than CRT. Consistent with these findings, studies in immunocompetent C57BL/6 mice with radioresistant B16-F10 melanoma revealed a fundamentally different immunomodulatory profile between MBRT and CRT [21]. MBRT robustly increased intratumoral CD8⁺ T cell and B cell infiltration while reducing regulatory T cell accumulation. In contrast, CRT not only failed to promote these beneficial immune changes but actually exacerbated the immunosuppressive state of the TME, with increased Treg infiltration. Mechanistically, MBRT upregulated the chemokine CXCL9 [21], a known attractant for effector T cells, which may explain its superior ability to recruit cytotoxic lymphocytes.

The temporal dynamics of immune activation further distinguish MBRT from conventional approaches. In immunocompetent F344 rats with RG2 glioma, MBRT achieved earlier recruitment of CD8⁺ and CD4⁺ T cells to the tumor core compared with CRT, while simultaneously causing less damage to normal brain tissue, a combination that defines a more favorable therapeutic window [14]. This observation raises an important question: can the immune benefits of MBRT be attributed primarily to its higher peak-to-valley dose ratio (PVDR)? Potiron et al. addressed this by matching valley doses between MBRT and CRT, revealing that MBRT induced significantly less infiltration of immunosuppressive Tregs and pro-inflammatory myeloid cells [80]. Their findings may suggest that higher dose heterogeneity—achieved by optimizing peak dose while maintaining a minimum valley dose—promotes a less immunosuppressive TME.

Collectively, these distinct immune effects support the combination of each SFRT modality with immunotherapy. However, the optimal combinatorial approach may vary substantially across different irradiation techniques and tumor subtypes.

#### Vascular remodeling and its immunological consequences

Tumor tissues continuously secrete high concentrations of proangiogenic factors, leading to the formation of a vascular network characterized by structural disorder and functional abnormalities within them [102–104]. Such malformed blood vessels can restrict the efficacy of various antitumor therapies through multiple pathways: on the one hand, they impede the infiltration of immune cells into tumor lesions. On the other hand, they limit the delivery efficiency of chemotherapeutic drugs into the tumor parenchyma; meanwhile, they attenuate the killing effect of RT on tumor cells [102–104]. Different RT modalities exhibit distinct regulatory effects on the tumor vascular system. Preclinical evidence indicates that conventional photon RT can stimulate tumor cells to secrete proangiogenic factors such as VEGF, and this stimulatory effect is dose-dependent; the higher the RT dose, the more pronounced the VEGF secretion by tumor cells [105–110]. Paradoxically, this phenomenon may accelerate tumor angiogenesis and even elevate the risk of tumor metastasis [105–110].

In contrast, preclinical animal studies have demonstrated that MRT exhibits unique and superior advantages in regulating tumor vasculature, with the sensitivity of blood vessels to MRT being highly dependent on their maturation stage [13]. Specifically, immature capillary plexuses are destroyed under MRT, whereas mature vascular structures display significant resistance to its effects [13]. Even when exposed to peak doses of several hundred Gy, MRT exerts minimal impact on the vascular network of mature blood vessels, and their structure, density, and perfusion remain intact [16–18, 111]. Notably, tumor blood vessels generally lack mature pericyte coverage and exhibit an abnormal basement membrane, and therefore predominantly display an immature phenotype both morphologically and functionally, making them a potentially preferential target of MRT [112–115]. For example, preclinical evidence from rodent models indicates that MRT preferentially disrupts immature tumor blood vessels, triggering a cascade of pathological changes including endothelial cell denudation, reduced vessel count, decreased perfusion, and subsequent tumor hypoxia [111, 116]. Based on these findings, it has been proposed that the selective cytotoxic effect of MRT on tumor vascular endothelial cells can eliminate dysfunctional, immature tumor blood vessels while preserving structurally intact, mature vessels, and that these preserved mature vessels may facilitate T-cell infiltration into the TME [117]. Should this mechanism be recapitulated in humans, it could further enhance the anti-tumor efficacy of SFRT while effectively preserving the structural and functional integrity of blood vessels in normal tissues. Clinical evidence directly linking MRT-induced vascular alterations to improved T cell infiltration remains limited to date. However, these models are overly simplified. CAM embryos and normal mouse models lack the complex biological mechanisms present in the TME and thus cannot faithfully recapitulate the vascular responses observed in clinical tumors. Furthermore, the observation dimensions are limited, with most studies neglecting long-term changes in vascular functional reserve and potential damage. Future research should be conducted in adult tumor-bearing mammalian models, combining long-term dynamic observations to systematically compare the differential sensitivity of MRT on tumor vasculature versus normal blood vessels.

In addition, preclinical studies have shown that MRT can downregulate VEGF expression in tumor tissues [85]. As a core regulatory factor in tumor angiogenesis, VEGF overexpression directly triggers vascular structural disorders, increased permeability and functional abnormalities. Therefore, downregulation of VEGF expression may facilitate the normalization of tumor blood vessels [118]. Further studies have revealed that after MRT treatment, the pericyte coverage rate (αSMA⁺) surrounding tumor blood vessels increases significantly. Based on these observations, it has been hypothesized that the structure of tumor blood vessels tends to be “normalized” by MRT [119]. However, it is important to note that this vascular normalization effect has been demonstrated only in preclinical MRT models (particularly with narrow-beam and high-dose MRT), and direct clinical validation is currently lacking. If confirmed in future studies, this phenomenon could markedly induce vascular normalization and tumor reoxygenation, thereby creating a therapeutic window for subsequent treatments including chemotherapy, immunotherapy, and re-irradiation. Dynamic MRI analyses of rats bearing intracranial F98 gliomas demonstrated that MRT accelerates and amplifies the enhancement of tumor vascular permeability compared to equivalent wide-beam RT. This accelerated disruption compromises the blood-tumor barrier, effectively priming a physical pathway for the transendothelial migration of effector immune cells into the tumor parenchyma [117]. Collectively, these findings suggest that MRT-mediated modulation of tumor vascular permeability can provide an adequate time window for the delivery of immunotherapeutic agents, implying that combining MRT with immunotherapy may be more effective than combining CRT with immunotherapy.

### Regional immune extension: the radiation-induced RIBE

The radiation-induced RIBE refers to biological responses in cells that are completely outside the radiation field and receive no direct irradiation, with effects mediated solely by signals from nearby irradiated cells. It has been proposed that the RIBE contributes to immune responses by enabling irradiated cells to transmit damage signals and pro-inflammatory cytokines to non-irradiated cells, thereby creating a pro-inflammatory microenvironment that facilitates the recruitment and activation of innate immune cells [120]. Robust evidence for RIBE comes from both preclinical and clinical studies. Clinical studies show that high-dose RT selectively targeting hypoxic tumor regions can significantly induce RIBE, with 96% of patients showing significant regression (median 70%) in non-irradiated tumor regions without treatment-related toxicity [121]. Preclinically, research has shown that GRID RT (with peak doses of 10 Gy and valley doses of ~ 1 Gy) resulted in significantly reduced cell survival in bystander regions compared to uniform 1 Gy irradiation [122]. T cells may play a critical role in mediating the RIBE and are likely derived from the irradiated tumor region [81]. Besides its effects on tumor cells, MRT also modulates blood vessels in non-irradiated regions. As reported by Fontanella et al. [123]., MRT upregulates HIF‑1 through bystander signaling, thereby increasing intravascular hemoglobin levels and promoting angiogenesis. This response may facilitate effector immune cell infiltration and the delivery of immunotherapeutic agents. Furthermore, studies using MRT in both F344 rats bearing 9 L gliosarcoma and tumor-free controls have also confirmed the presence of RIBE in the non-irradiated areas [15]. In a rat C6 glioma model in vivo, Fernández‑Palomo et al. detected spreading γ-H2AX signals from MRT high-dose peaks to valley regions via staining [124]. They hypothesized that peak zones induced DNA damage in valley cells via a unique “bystander-like effect” that differs from conventional RIBE. However, the authors acknowledged that since the actual physical dose in valleys was not quantified, the relative contributions of bystander-like signaling and scattered radiation could not be distinguished. Therefore, this phenomenon remains speculative and requires independent validation. Using in vitro human cancer cell models, Pakniyat et al. investigated the influence of high-dose peak regions on valley cells during GRID RT [125]. They showed that cells in GRID-shielded regions (receiving ~ 1.5 Gy) showed significantly higher DNA damage and micronucleus formation than cells exposed to uniform 1.5 Gy irradiation. These findings indicated a bystander-like effect, which was more pronounced in radioresistant tumor cells. However, these findings lack validation from in vivo studies, and whether similar responses occur in animal models or human patients is unknown.

The underlying mechanism of RIBE may involve multiple intercellular signaling pathways. The primary process begins with the release of signaling molecules from irradiated tumor cells [126]. These molecules can be transmitted to neighboring bystander cells through direct cell-to-cell contact or gap junctions [127]. Subsequently, this signal transmission triggers a series of biological responses in bystander cells, including activation of DNA repair mechanisms, cell cycle arrest, and upregulation of antioxidant gene expression [122, 127]. The signaling molecules mediating RIBE may include nitric oxide (NO), ROS, TNF-α, TGF-β1, and microRNAs (miRNAs), among others [15, 128–131]. Notably, in certain scenarios, such as alpha-particle radiation, a distinct mitochondrial transfer mechanism operates. Using LLC cells and a nude mouse xenograft model, it was demonstrated that damaged mitochondria can be transferred from irradiated cells to bystander cells [94, 132]. This process may lead to increased ROS levels in recipient bystander cells, thereby mediating DNA double-strand breaks and clustered oxidative damage, ultimately inducing significant biological effects in low-dose tissues [94, 132]. However, the relative contribution of each signaling pathway under SFRT remains unclear. The immunomodulatory effects of SFRT might be partly attributable to radiation-induced bystander signaling. The secretion of pro-inflammatory cytokines and DAMPs by irradiated cells could potentially transmit damage signals to neighboring non-irradiated cells, which in turn may create a pro-inflammatory milieu that recruits and activates innate immune populations. This signaling cascade has been hypothesized to facilitate the transition from innate to adaptive immunity. However, direct experimental evidence linking RIBE to adaptive immune responses in the context of SFRT remains lacking. It is important to emphasize that most RIBE-related findings in SFRT originate from in vitro models and specific preclinical platforms such as MRT studies performed in rodent brains, and their relevance to human SFRT remains speculative at present. Whether bystander signaling acts as a direct trigger for immune activation or instead functions as a parallel, independent pathway remains a critical avenue for future investigation.

### Systemic immune control: activating the abscopal effect

Beyond regional RIBE, anti-tumor effects occurring outside the local RT field, at sites distant from the radiation area and not caused by scattered radiation, are defined as the abscopal effect [7, 133]. A large number of clinical case reports have documented the occurrence of the abscopal effect during RT, including in diseases such as chronic lymphocytic leukemia, malignant lymphoma, papillary adenocarcinoma, and liver cancer [134–139].

Further studies in animal models have reported that, in certain experimental settings (e.g., bilateral LLC tumors treated with LRT), the abscopal effect induced by specific SFRT modalities achieved greater tumor control than CRT. In a representative model of bilateral LLC tumor-bearing mice, one tumor lesion received a single 20 Gy radiation dose. Results showed that LRT irradiation of only 20% of the tumor volume (divided into two 10% apical regions) not only effectively delayed local tumor growth but also significantly inhibited distant non-irradiated tumor growth (i.e., the abscopal effect). Conversely, CRT failed to suppress distant metastases, despite achieving robust local tumor control [7]. Similarly, in immunocompetent BALB/c mouse models of 4T1 triple-negative breast cancer, comparative studies between GRID RT and CRT demonstrate that GRID RT can significantly delay the growth of abscopal tumors, accompanied by T cell infiltration and elevated IFN-γ levels in abscopal tumors [97]. Nevertheless, the magnitude of this effect likely varies across distinct tumor types and SFRT configurations. Moreover, direct comparisons pitting SFRT against CRT to evaluate systemic efficacy are scarce and restricted to limited preclinical data.

As suggested in preclinical studies, the induction of the abscopal effect by RT is closely associated with immune system activation, with T lymphocyte infiltration serving as a central mechanism [133, 140–142]. In F98 glioma-bearing nude mice, a model characterized by severe T-cell deficiency but intact innate immunity, localized whole-brain MRT unexpectedly triggered systemic responses in distal bladder tissues, proving that the abscopal effect can bypass classical adaptive immune pathways [143]. Subsequent studies using MRT verified this hypothesis using three immune-deficient models: NOD scid gamma (NSG) mice (lacking T, B, and NK cells), macrophage-depleted mice treated with anti-colony-stimulating factor 1 receptor (anti-CSF-1R) antibodies, and CCL2-knockout mice (with impaired macrophage chemotaxis). Lobachevsky et al. hypothesized that the abscopal effect induced by localized irradiation requires an intact adaptive immune system and is critically dependent on CCL2-mediated macrophage recruitment and activation [144]. Nevertheless, there remains a lack of conclusive evidence to clarify the specific molecular pathways underlying SFRT-induced abscopal effects.

In all, clinical investigations remain limited in elucidating the underlying antitumor mechanisms of SFRT; most relevant studies only report and verify clinical therapeutic efficacy. Insights into its core antitumor mechanisms are still predominantly derived from preclinical basic research. Recent preclinical studies increasingly suggest that the efficacy of SFRT may extend beyond direct tumor cell killing and its deeper value may lie in its ability to reshape the TIME. This framework could provide a theoretical basis for understanding the complex phenomena observed preclinically, and might also offer inspiration for future research aimed at explaining those sporadic yet impressive clinical success stories. Focusing on dose heterogeneity, two preclinical studies using MBRT have suggested that valley dose, rather than peak dose, shows the strongest correlation with tumor response [79], and that greater dose heterogeneity (achieved by optimizing peak dose while maintaining a low valley dose) promotes the formation of a less immunosuppressive TME [80]. These findings indicated that when investigating the immune effects of MBRT, peak and valley doses may need to be considered as an integrated whole, as they shape the ultimate immunological outcome. However, whether similar dose‑heterogeneity principles apply to other SFRT techniques (e.g., GRID or LRT) remains an open question. In exploring the immunological mechanisms of SFRT, studies across various tumor models and SFRT modalities have frequently reported that SFRT increases T cell infiltration [7, 21, 81, 83, 85, 86, 97, 99]. Specifically, MRT and MBRT are associated with increased infiltration of CD8⁺ T cells and B cells as well as reduced Treg populations [21, 83, 85, 86]; LRT elevates CD3⁺ T cell levels in both irradiated and non-irradiated tumors [7]; and GRID RT is correlated with upregulated PD-L1 expression in abscopal lesions [97]. Beyond changes in immune cell populations, dynamic alterations in the cytokine network [83, 94] indicate that SFRT triggers a systemic anti-tumor response mediated by intricate cell-cytokine interactions. Cytokine changes have been largely explored in MRT models, and whether similar patterns occur with other SFRT platforms remains less well defined. Furthermore, preclinical studies on MRT have reported its preferential effects on tumor vasculature, induction of vascular normalization [13, 119], and alterations in vascular permeability [114, 115], all of which may facilitate the delivery of immunotherapeutic agents to tumor sites. It should be emphasized that these vascular effects have been primarily documented in MRT; evidence from GRID, LRT, or MBRT is currently lacking. Both preclinical and clinical studies have suggested that GRID RT and MRT could act on cells in adjacent unirradiated tumor regions via the RIBE, although its underlying molecular mechanisms remain unclear and speculative. Of greater importance, within the same irradiated target volume, certain SFRT techniques may also trigger the bystander-like effect. Nevertheless, the precise mechanisms underlying this effect remain hypothetical and require extensive and well-designed studies for further validation and exploration. Notably, control of unirradiated distant tumors, namely the abscopal effect, has been observed in both preclinical and clinical settings [71, 143]. This finding is significant. It suggests that the immunomodulatory effects of SFRT are not confined to the irradiated field but may also inhibit distant metastatic tumors by activating systemic anti-tumor immunity. Finally, if a given SFRT modality can systemically recruit effector T cells to both primary and distant tumors [143], then ICIs should theoretically amplify and sustain this response.

## Synergistic integration of SFRT and immunotherapy

### Preclinical evidence of SFRT in combination with immunotherapy

Several preclinical studies have suggested that the combination of SFRT and immunotherapy may exhibit synergistic effects in inducing tumor regression (Table 2). These findings provide a preliminary theoretical foundation for the future clinical application of SFRT, particularly in combination with immunotherapy. However, the evidence base is heterogeneous, and critical gaps of the underlying mechanisms and optimal combination strategies remain.

**Table 2:** Efficacy of spatially fractionated radiotherapy (SFRT) in combination with immunotherapy in preclinical models and clinical cases

Ref.	Model/Tumor	Tumor Volume	SFRT Type	Immunotherapy (Outcomes)	Study Design	Immune Monitoring	Clinical Status
[150]	F344 (9 L)	NA	MRT	GMIMPR: >40% of rats survived > 1 year without disability (vs. MRT alone)	NA	NA	Preclinic
[149]	C57BL/6 (MOC1 OSCC, MOC1-HPV K1)	NA	pIR	Anti-PD-1: Longer survival (vs. pIR alone) in MOC1 model; pIR gave lower tumor growth inhibition than CRT	NA	NA	Preclinic
[97]	Balb/c (4T1)	NA	GRID	Anti-PD-1 + Anti-CTLA-4: Upregulated PD-L1 in primary tumor; suppressed primary and distant tumor growth; increased CD8⁺ T cells in distant tumors and elevated IFN-γ in blood	NA	NA	Preclinic
[21]	C57BL/6 (B16-F10)	NA	MBRT	Anti-CTLA-4: Longer survival (vs. CRT+anti-CTLA-4); long-lasting anti-tumor memory; CD8⁺ T cell depletion abrogates tumor control	NA	NA	Preclinic
[22]	C57BL/6 (MC38); BALB/c (CT26, 4T1)	NA	pIR	Anti-PD-1: ~90% CR in CT26; extended survival in MC38 and 4T1; adding CXCR2 inhibitor in MC38 further improved tumor control (> 50% CR)	NA	NA	Preclinic
[147]	C57BL/6 (B16-F10)	NA	MRT	Anti-PD-1 + Anti-CTLA-4: 40% CR and long-term survival (not achieved with MRT alone); abscopal responses	NA	NA	Preclinic
[145]	C57BL/6 (EO771)	NA	MBRT	Anti-PD-L1: Stronger abscopal effect (vs. CRT); higher percentage of CD8⁺ T cells systemically and at the abscopal tumor site (vs. CRT)	NA	NA	Preclinic
[146]	F344 (RG2)	NA	MBRT	Anti-PD-L1: No significant survival benefit over MBRT alone	NA	NA	Preclinic
[152]	NSCLC (PD-L1 > 70%)	63.2 cm³	LRT	Anti-PD-1(Pembrolizumab): CR in treated lesion; other lesions PR; CR maintained at 5 months	Case report	No	Clinic
[59]	Melanoma (resistant to multiple immunotherapies)	650 cm³	GRID	Anti-PD-1(Pembrolizumab): 75% tumor regression at 2 months and CR at 5 months; retroperitoneal disease stable for 6 months; neck disease-free at 12 months	Case report	No	Clinic
[151]	Metastatic melanoma	12.0 × 9.1 cm	GRID	Anti-PD-1་CTLA-4(Nivolumab + Ipilimumab): Complete pathologic response at 5 months; disease-free for 2.5 years; grade 3 hepatitis and grade 2 rash (resolved)	Case report	No	Clinic
[153]	HCC (anti-PD-1 refractory)	12.8 × 7.0 cm	LRT	Anti-PD-1/CTLA-4 (Cadonilimab): 40% tumor regression at 2 months; PFS 5 months; OS 1 year; no grade 3/4 toxicity	Case report	No	Clinic
[154]	Cervical cancer with multisite metastasis	6.6 cm	LRT	Anti-PD-1/CTLA-4 (Cadonilimab): Halted cadonilimab-driven rapid tumor progression	Case report	No	Clinic

Abbreviations: HCC = hepatocellular carcinoma; EGFR = epidermal growth factor receptor; PR = partial response. Reference numbers correspond to the citations in the main text. Preclinical studies of SFRT combined with immunotherapy; clinical cases of SFRT plus ICIs in solid tumors are from the past decade

In the murine glioma model, MBRT alone failed to achieve durable anti-tumor effects. However, when combined with anti-cytotoxic T-lymphocyte-associated antigen 4 (anti-CTLA-4) therapy, it resulted in significantly prolonged survival compared to CRT combined with the same immunotherapy, and induced a long-lasting anti-tumor memory response [21]. While this finding compellingly illustrates the potential of MBRT to synergize with immunotherapy, the study’s design leaves critical questions unanswered. It did not explore the optimal sequencing of immunotherapy relative to MBRT (i.e., before or after radiation) or the ideal interval between MBRT and ICI administration to maximize the anti-tumor response. Furthermore, in an EO771 breast cancer mouse model, MBRT (50 Gy peak dose) combined with anti-PD-L1 was more effective in controlling both primary and distant tumors than uniform RT (10 Gy) combined with anti-PD-L1, which suggested a possible association with an increase in the number and activation status of CTLs in both non-irradiated distant tumors and the spleen [145]. However, the promise of MBRT-ICI combinations is tempered by contradictory findings. In an orthotopic rat glioma model, Iturri et al. found that addition of anti-PD-L1 did not significantly improve outcomes compared to MBRT alone [146]. This discrepancy with other positive reports may be attributed to methodological differences, including the use of a distinct, potentially more immunosuppressive tumor model (glioma vs. melanoma or breast cancer), variations in the average radiation dose delivered, and critically, the timing of ICIs administration, which can profoundly influence T-cell priming and infiltration. These conflicting results highlight the urgent need for systematic optimization of treatment parameters across different tumor types before clinical translation.

Moreover, in a murine melanoma model, MRT was observed not only to induce a potent local anti-tumor immune response characterized by enhanced antigen presentation and substantial infiltration of CTLs, but also to trigger a systemic abscopal effect, controlling locoregional metastases [147]. Crucially, direct testing of MRT combined with dual ICIs revealed a clear synergistic effect, yielding a 40% rate of complete tumor regression and long-term survival, an outcome not achievable with MRT alone [147]. However, this study lacked a control group receiving combined CRT and ICIs, so it cannot directly quantify the specific therapeutic advantage of MRT over CRT. In other studies utilizing murine models of breast and colorectal cancer, pIR (delivering 16 Gy and 2 Gy to different parts of the same tumor, respectively) was implemented in combination with anti-PD-1 antibody [22]. This combination therapy was associated with remodeling of CTLs toward a more active state with an interferon-activated phenotype, leading the authors to propose a potential mechanism by which pIR may synergize with and enhance the efficacy of anti-PD-1 therapy [22]. They also identified C-X-C motif chemokine receptor 2 (CXCR2) as a potential target for reducing the infiltration of pro-tumor neutrophils following pIR. Combining a CXCR2 antagonist (SB225002) with PD-1 blockade after pIR further improved tumor control and mouse survival [22]. In this study, the immunomodulatory advantages of intentional dose heterogeneity have been further demonstrated using high-dose-rate brachytherapy to create a steep intratumoral dose gradient (2–20 Gy) in melanoma and prostate cancer models [148]. Heterogeneous distribution primed the TME, enhancing the response to dual ICIs and inducing systemic anti-tumor immunity, including growth inhibition of distant, non-irradiated tumors [148]. The antitumor effect in this model required both CD4^+^ and CD8^+^ T cells and was associated with spatial heterogeneity in gene expression and clonal expansion of effector CD8^+^ T cells [148]. Although pIR differs from conventional SFRT, the study’s findings suggest that heterogeneous intratumoral dosing combined with ICIs may elicit systemic immune responses. In immunocompetent 4T1 breast cancer mouse models, GRID RT combined with ICIs upregulated PD‑L1 expression in primary tumors, suppressed primary tumor growth, and distant non‑irradiated tumor growth [97]. These responses were accompanied by increased CD8⁺ T cell infiltration in distant non‑irradiated tumors and elevated IFN‑γ levels in peripheral blood, suggesting that GRID RT combined with immunotherapy may activate systemic anti‑tumor immune responses [97]. This study provides supportive evidence that the unique dosimetry of GRID RT may translate into superior therapeutic outcomes compared to CRT [97]. However, the synergy between SFRT and ICIs is not universal. For example, pIR did not demonstrate superior efficacy to whole-tumor radiotherapy (WTRT) in enhancing the effectiveness of ICIs [149]. Although pIR combined with anti-PD-1 prolonged survival compared to pIR alone in the MOC1 model, this benefit was accompanied by only a modest increase in CTL infiltration and was absent in the MOC1-HPV K1 model. Responding tumors were enriched with effector memory T cells, whereas non-responders exhibited increased infiltration of MDSCs and monocyte-derived DCs [149]. These findings suggest that the pre-existing TME may shape therapeutic outcomes. The synergy between SFRT and ICIs could be substantially constrained in tumors characterized by low baseline immunogenicity and a dominant immunosuppressive myeloid infiltrate, necessitating further optimization of irradiation parameters and dosing schedules, as well as the addition of agents that can remodel the “cold” TME.

Beyond passive immunotherapy, MRT has also been investigated in combination with active immunotherapy. Comparative studies in murine EMT6.5 breast cancer models revealed that MRT induces a gene expression profile fundamentally distinct from that of CRT; notably, it exhibits an attenuated capacity to induce the key chemokine GM-CSF [87]. To compensate for this inherent deficiency, preclinical investigations explored the effect of combining MRT with gene-mediated immunoprophylaxis (GMIMPR), which uses genetically engineered tumor cells to secrete GM-CSF and prime DCs. In a rat 9 L gliosarcoma model, the combination of MRT and GMIMPR effectively enhanced tumor-specific cytotoxic T-cell responses by supplementing MRT’s insufficient endogenous GM-CSF induction with exogenous GM-CSF, thereby markedly suppressing tumor growth [91, 150]. However, it is important to note that these findings are derived from specific tumor models and may not generalize across all tumor types or SFRT modalities. The immune-modulating effects of different SFRT techniques (GRID, LRT, MBRT, MRT) or different parameters of the same technique (PVDR, dose rate) are likely to induce distinct changes in the immune microenvironment, and the optimal parameters for achieving synergistic effects with immunotherapy remain to be defined. Future research should systematically map the immune modulation atlas across different SFRT techniques (e.g., cytokine profiles, immune cell infiltration dynamics).

Collectively, these preclinical evidence suggests that combining SFRT with immunotherapy may achieve local tumor control and, in some models, trigger systemic anti-tumor immune responses that can suppress distant metastases. However, the magnitude and durability of such responses vary considerably according to tumor type, baseline immune contexture, and SFRT parameters. Therefore, while these findings provide a rationale for further investigation, they do not yet support the claim of a ready-to-use clinical strategy. Further validation in more diverse preclinical models and, ultimately, in well-designed clinical trials is required before this approach can be translated into the clinical management of advanced metastatic disease.

### Clinical evidence of SFRT in combination with immunotherapy

Beyond the preclinical models, some preliminary clinical evidence also supports the potential of this combination. Mohiuddin et al. documented complete regression of a large melanoma mass (18 × 15 × 8 cm) in a patient with dual resistance to ipilimumab and pembrolizumab following a single 20 Gy fraction of GRID RT combined with pembrolizumab [59]. This case suggests that GRID RT may overcome established ICI resistance, likely through immune‑mediated mechanisms. This case compellingly illustrates that GRID RT can overcome established resistance to multi-agent immunotherapy, likely through immune-mediated mechanisms [59]. Although no clinical studies have yet confirmed the specific mechanisms by which combination therapy reverses immunotherapy resistance, preclinical evidence suggests that GRID RT treatment increases the infiltration of activated CD8⁺/CD4⁺ T cells and DCs into both irradiated and distant tumors, elevates IFN-γ levels, and upregulates PD-L1 expression in abscopal lesions, thereby creating a TME more susceptible to ICIs [97]. Building on this, a more recent case report by Hobson et al. described a patient with advanced melanoma who, after progression on dual ICIs (Nivolumab + Ipilimumab), achieved a complete pathologic response following treatment with GRID RT combined with the same dual ICIs regimen [151].

LRT has provided the most compelling clinical evidence that its efficacy is predominantly immune‑mediated. Jiang et al. performed an intra‑patient comparison in a patient with metastatic non-small cell lung cancer (NSCLC) receiving continuous anti‑PD‑1 therapy [152]. While multiple metastatic lesions were treated with various palliative regimens (including CRT, SBRT, and low‑dose LRT), only the patient treated with high‑dose LRT (20 Gy peak dose, covering only 6.5% of the gross tumor volume) for a posterior chest wall mass achieved a CR and remained disease‑free until the patient’s death seven months later [152]. The calculated effective uniform dose for this lesion was only 1.2 Gy, which would be insufficient to achieve local control according to conventional radiobiological principles. Nevertheless, the lesion regressed completely, which strongly suggested a synergistic interaction between high-dose LRT and anti-PD-1 blockade and cannot be attributed to direct tumoricidal effects alone [152]. This intra-patient finding echoes preclinical studies in bilateral LLC tumor models, where LRT simultaneously enhanced CD3⁺ T cell infiltration in both irradiated and non-irradiated tumors, whereas whole-tumor irradiation failed to elicit such systemic effects [7]. This finding suggests that LRT can reshape the TIME and may convert tumors into “hot” tumors with extensive T-cell infiltration, thus providing a key target for ICIs [7]. Additionally, Hu et al. reported a case of a growing hepatocellular carcinoma that had failed prior anti-angiogenesis and anti-PD-1 therapy. However, the addition of LRT to ongoing immunotherapy led to disease control [153]. Similarly, Liu et al. described a case of cervical cancer with multisite metastasis treated with LRT combined with cadonilimab (a PD-1/CTLA-4 bispecific antibody), reporting favorable outcomes [154]. While these cases extend the potential applicability of LRT-ICI combinations to other tumor types, they lack the definitive intra-patient control.

In addition to the classical SFRT modalities discussed above, several related unconventional RT approaches have been explored for their potential to synergize with immunotherapy. A phase 2 study by Tubin et al. performed a study involving 60 patients with unresectable bulky NSCLC. The trial compared SBRT-based partial tumor irradiation of the hypoxic segment (SBRT-PATHY), an irradiation strategy targeting hypoxic tumor cores and protecting peritumoral immune microenvironment, against CRT and chemotherapy [155]. SBRT-PATHY achieved significantly higher survival and tumor control rates, induced bystander and abscopal effects in most patients, and was associated with lower toxicity. Multivariate analysis confirmed SBRT-PATHY as an independent predictor of improved survival. Building on this concept, Massaccesi et al. reported a clinical case of a patient with renal cell carcinoma and peritoneal metastases refractory to a PD-1 inhibitor. The patient received a novel immune-sparing partial ablative radiation therapy (ISPART) [156]. No acute side effects were reported, only transient pain exacerbation one day after the last ISPART session, which resolved spontaneously [156]. A systemic CE-CT scan two months later showed a 30% reduction in the target lesion. This case supports the synergistic effect of ISPART with nivolumab [156]. Although SBRT-PATHY and ISPART are not classic examples of SFRT, both of them employ a core strategy shared with SFRT: partial-volume rather than whole-tumor homogeneous irradiation. The favorable outcomes of these two unconventional RT modalities raise the possibility that the therapeutic efficacy of spatially modulated dose distribution strategies may not strictly depend on a specific technical platform (e.g., GRID, LRT, MBRT, MRT), but rather on the fundamental physical principle that underlies all of them, namely the creation of a spatially modulated dose distribution.

A parallel comparison of the evidence presented in Table 2 yields several broader insights that transcend individual studies. These insights directly point to the core limitations of the current field and highlight key directions for future research. Consistent with the broader SFRT literature, clinical evidence regarding SFRT plus immunotherapy mainly comes from case reports. Such findings remain hypothesis-generating and are susceptible to confounding variables, including concurrent systemic treatment, selection bias, and a lack of systematic immune correlative analyses. To date, no prospective randomized controlled trials have been published. At present, there is no agreement on the best timing to combine immunotherapy with specific SFRT modalities (such as GRID, LRT, MBRT, MRT), whether in preclinical or clinical studies. While some preclinical models have shown no significant difference between sequential and concurrent administration [157], clinical findings remain divergent-certain studies suggest that initiating immunotherapy immediately after RT may improve prognosis [158, 159], whereas others support pre-RT administration [160] or report no significant impact of treatment sequence on efficacy [161]. These inconsistencies suggest that the optimal timing for SFRT-immunotherapy combinations is unlikely to be determined by a single variable, but rather modulated by multiple interacting factors, and may differ across SFRT techniques. The divergent outcomes of MBRT across different tumor models further illustrate this complexity. While MBRT demonstrated remarkable efficacy in melanoma [22] and breast cancer [145], it showed limited benefit in glioma [146]. Such differences mainly stem from the baseline heterogeneity of the TIME. Specifically, “cold” tumors such as glioma are inherently deficient in constitutive T cell infiltration, and even with the optimal radiation dose, this will greatly limit antigen release and subsequent CD8 + T cell activation, making it difficult to trigger a potent anti-tumor immune response and thus potentially reducing the efficacy of combination therapy. Several preclinical studies using different SFRT modalities (GRID, MRT, pIR, and GMIMPR combined with MRT) suggest that in their respective models, these interventions all could exert a synergistic effect with immunotherapy, thereby achieving local tumor control and triggering abscopal effects [97, 147, 149, 150]. Separately, clinical case reports have indicated that GRID RT reversed immunotherapy resistance in melanoma [59] and that LRT achieved complete remission in NSCLC [152], an outcome rarely achievable with CRT alone. While these clinical observations align with the immunomodulatory potential suggested by preclinical studies, direct mechanistic validation is currently lacking. Furthermore, diverse combination regimens incorporating different SFRT modalities have not been systematically verified in prospective large-scale clinical trials to date.

In view of the above controversies and research gaps, future studies should focus on individualization and precision. The core tasks are to develop and validate predictive biomarkers and clarify the optimal sequence of immunotherapy. The SBRT-PATHY design [155], which targets the hypoxic tumor core, suggests that subsequent SFRT research can optimize RT modalities with a focus on dose targeting. Patient-specific factors, including immune status, tumor burden, and prior treatment history, should be systematically controlled to reduce clinical heterogeneity.

## Discussion

The translation of SFRT-ICI combinations from occasional salvage therapy into a precision immuno-radiotherapy paradigm hinges on a coordinated, multi-level research roadmap. At the preclinical level, systematic optimization of SFRT parameters is essential to maximize immune activation. This demands factorial experimental designs across tumor models with distinct immune phenotypes to determine the optimal peak dose, valley dose, fractionation, and most critically, the sequence and interval of ICIs administration relative to SFRT. Concurrently, tumor type-specific mechanisms of resistance and response warrant investigation, establishing dose-response curves based on immune endpoints (T cell infiltration, cytokine induction, PD-L1 upregulation) rather than tumor control alone. Humanized mouse models and patient-derived xenografts should complement traditional syngeneic models to better recapitulate the complexity of the human TIME. Equally important, continued preclinical efforts should map the “immune modulation atlas” of different SFRT techniques. Distinct SFRT modalities (GRID, LRT, MBRT, MRT) and variations in their technical parameters (PVDR, beam width, vertex spacing, fractionation) may drive distinct cytokine signatures and temporal alterations in immune cell infiltration. Understanding these differences may therefore provide a theoretical basis for translating SFRT into clinically relevant research paradigms. Additionally, the GMIMPR paradigm, which identifies MRT’s GM-CSF deficiency and pairs it with GMIMPR to enhance tumor-specific T cell responses, exemplifies a “mechanism-driven pairing” approach that could inform future SFRT-ICI combination design. Based on preclinical observations that “cold” tumors fail to benefit from SFRT-ICI, baseline tumor immune contexture (including T cell infiltration, MDSC frequency, myeloid populations) should be prospectively correlated with SFRT-ICI response. Serial monitoring of circulating immune cells, cytokine profiles, and ctDNA dynamics may identify early predictors of response and resistance. On the clinical front, prospective phase II/III trials with modality-specific standardized SFRT parameters and comprehensive immune monitoring are essential. These trials must incorporate serial tumor biopsies and peripheral blood sampling to validate whether preclinical mechanisms, including T cell infiltration, cytokine shifts, and PD-L1 upregulation, operate in patients. Critically, trial designs must address specific questions regarding ICI sequencing and timing, comparing concurrent versus sequential administration and varying intervals between SFRT and ICIs initiation.

The ultimate goal is to transform SFRT-ICI combinations from empirical palliation into precision immuno-RT. As these gaps are progressively bridged, SFRT-ICI combinations could not only debulk bulky disease but also activate durable systemic immunity capable of eradicating occult metastases. With more comprehensive investigation, SFRT can maximally engage the immune system, extending therapeutic benefits systematically to more patients, more tumor types, and against micrometastatic disease—this is both the challenge we face and the opportunity that lies ahead.

## Conclusions

In conclusion, the distinct spatial dose distribution of SFRT may help create a tumor microenvironment conducive to systemic immune activation, providing a strong rationale for combining SFRT with immunotherapy. Although current clinical evidence remains preliminary and largely hypothesis-generating, the accumulating preclinical foundation supports the concept that SFRT is not merely a cytoreductive tool but a promising systemic immunomodulatory modality. Further rigorous investigation, including optimized SFRT parameters and well-designed clinical trials, is warranted to translate this strategy into clinical practice.

## Data Availability

No datasets were generated or analysed during the current study.

## Abbreviations

ATP: Adenosine triphosphate
CCL2: C-C motif chemokine ligand 2
CCL5: C-C motif chemokine ligand 5
cGAS-STING: Cyclic GMP-AMP synthase-stimulator of interferon genes
CRT: Conventional radiotherapy
CTL: Cytotoxic T lymphocyte
CTLA-4: Cytotoxic T-lymphocyte-associated protein 4
CXCL9: C-X-C motif chemokine ligand 9
CXCR2: C-X-C motif chemokine receptor 2
DAMP: Damage-associated molecular pattern
DC: Dendritic cell
GM-CSF: Granulocyte-macrophage colony-stimulating factor
GRID: Grid radiotherapy
GTV: Gross tumor volume
HDLRT: High-dose lattice radiotherapy
HMGB1: High mobility group box 1
HSP: Heat shock protein
ICD: Immunogenic cell death
ICI: Immune checkpoint inhibitor
IFN-β: Interferon-beta
TNF‑α: Tumor necrosis factor-alpha
IFN-γ: Interferon-gamma
IL: Interleukin
ISPART: Immune sparing partial ablative radiation therapy
LD-RT: Low-dose radiotherapy
LRT: Lattice radiotherapy
MBRT: Minibeam radiotherapy
MDC: Macrophage-derived chemokine
MDSC: Myeloid-derived suppressor cell
MHC-I: Major histocompatibility complex class I
MCP-1: Monocyte chemoattractant protein‑1
MIP-1α: Macrophage inflammatory protein-1 alpha
MRT: Microbeam radiotherapy
NSCLC: Non-small cell lung cancer
NK cell: Natural killer cell
PD-1: Programmed cell death protein 1
PD-L1: Programmed death-ligand 1
PD1-IL2v: Programmed Death 1-Interleukin 2 variant
PDAC: Pancreatic ductal adenocarcinoma
PVDR: Peak-to-valley dose ratio
RIBE: Radiation-induced bystander effect
ROS: Reactive oxygen species
RT: Radiotherapy
SBRT: Stereotactic body radiotherapy
VMAT: Volumetric modulated arc therapy
EBRT: External beam radiotherapy
PATHY: Partial tumor irradiation targeting hypoxic segment
SIB: Simultaneous integrated boost
SFRT: Spatially fractionated radiation therapy
TAA: Tumor-associated antigen
TAM: Tumor-associated macrophage
TGF-β: Transforming growth factor-beta
TIL: Tumor-infiltrating lymphocyte
TIMP1: Tissue inhibitor of metalloproteinase 1
TME: Tumor microenvironment
TIME: Tumor immune microenvironment
TNF-α: Tumor necrosis factor-alpha
Treg: Regulatory T cell
VEGF: Vascular endothelial growth factor
WTRT: Whole tumor radiotherapy
TIL: Tumor-infiltrating lymphocyte
Gy: Gray
GMIMPR: Gene‑mediated immunoprophylaxis
